# Supplementary material for: Rapid modulation of striatal cholinergic interneurons and dopamine release by satellite astrocytes
Source: Nat Commun. 2024 Nov 19;15:10017. doi: 10.1038/s41467-024-54253-7 (PMC11577008; doi:10.1038/s41467-024-54253-7)
Supplement: Supplementary file 2 — Reporting Summary [file 41467_2024_54253_MOESM2_ESM.pdf]

nature portfolio

Corresponding author(s): Prof Stephanie Cragg

Last updated by author(s): Oct 21, 2024

Reporting Summary

Nature Portfolio wishes to improve the reproducibility of the work that we publish. This form provides structure for consistency and transparency in reporting. For further information on Nature Portfolio policies, see our Editorial Policies and the Editorial Policy Checklist.

Statistics

For all statistical analyses, confirm that the following items are present in the figure legend, table legend, main text, or Methods section.

n/a	Confirmed	
☐	☒	The exact sample size (*n*) for each experimental group/condition, given as a discrete number and unit of measurement
☐	☒	A statement on whether measurements were taken from distinct samples or whether the same sample was measured repeatedly
☐	☒	The statistical test(s) used AND whether they are one- or two-sided<br>*Only common tests should be described solely by name; describe more complex techniques in the Methods section.*
☒	☐	A description of all covariates tested
☐	☒	A description of any assumptions or corrections, such as tests of normality and adjustment for multiple comparisons
☐	☒	A full description of the statistical parameters including central tendency (e.g. means) or other basic estimates (e.g. regression coefficient) AND variation (e.g. standard deviation) or associated estimates of uncertainty (e.g. confidence intervals)
☐	☒	For null hypothesis testing, the test statistic (e.g. F, t, r) with confidence intervals, effect sizes, degrees of freedom and P value noted<br>*Give P values as exact values whenever suitable.*
☒	☐	For Bayesian analysis, information on the choice of priors and Markov chain Monte Carlo settings
☒	☐	For hierarchical and complex designs, identification of the appropriate level for tests and full reporting of outcomes
☒	☐	Estimates of effect sizes (e.g. Cohen's *d*, Pearson's *r*), indicating how they were calculated

Our web collection on statistics for biologists contains articles on many of the points above.

Software and code

Policy information about availability of computer code

Data collection	All original code used for anatomical modelling and analysis of electrophysiology data has been deposited in Zenodo (DOI:10.5281/zenodo.10553376).
Data analysis	Publicly available

For manuscripts utilizing custom algorithms or software that are central to the research but not yet described in published literature, software must be made available to editors and reviewers. We strongly encourage code deposition in a community repository (e.g. GitHub). See the Nature Portfolio guidelines for submitting code & software for further information.

Data

Policy information about availability of data

All manuscripts must include a data availability statement. This statement should provide the following information, where applicable:

- Accession codes, unique identifiers, or web links for publicly available datasets
- A description of any restrictions on data availability
- For clinical datasets or third party data, please ensure that the statement adheres to our policy

All data will be made available prior to publication through an open access data deposit. (Zenodo link)

Research involving human participants, their data, or biological material

Policy information about studies with human participants or human data. See also policy information about sex, gender (identity/presentation), and sexual orientation and race, ethnicity and racism.

Reporting on sex and gender	Some examplar data included from observations in post-mortem tissue from human brains of 3 males. Findings intended to validate observations from mouse. Tissue from females was not made available at time of study.
Reporting on race, ethnicity, or other socially relevant groupings	Not relevant.
Population characteristics	Age at death 70-82 years
Recruitment	Post-mortem human tissue samples were collected in accordance with approved protocols by the Ethics Committee of the University of Oxford (ref 15/SC/0639). All participants had given prior written informed consent for the brain donation.
Ethics oversight	University of Oxford

Note that full information on the approval of the study protocol must also be provided in the manuscript.

Field-specific reporting

Please select the one below that is the best fit for your research. If you are not sure, read the appropriate sections before making your selection.

[x] Life sciences [ ] Behavioural & social sciences [ ] Ecological, evolutionary & environmental sciences

For a reference copy of the document with all sections, see nature.com/documents/nr-reporting-summary-flat.pdf

Life sciences study design

All studies must disclose on these points even when the disclosure is negative.

Sample size	Sample sizes were chosen based on previous publications using similar approaches by this lab for either fast-scan cyclic voltammetry, patch-clamp electrophysiological recordings, or anatomy. All procedures on animals were conducted in accordance with the Animals (Scientific Procedures) Act 1986 (United Kingdom), approved by an Animal Welfare and Ethical Review Board at the University of Oxford, under the authority of Licenses from the Home Office (UK) (Numbers P9371BF54 and PP8860348).
Data exclusions	Data exclusions were not performed for any stage beyond initial recording and quality control
Replication	All results were obtained from at least two independent cohorts yielding similar results
Randomization	Randomization was performed on selecting cells for spatial analyses, for selecting regions for recording DA release kinetics.<br>For physiology and other live recordings, cells/regions/experiments to be used were selected or ordered at random.
Blinding	No blinding was performed for optogenetic or drug application experiments for fast-scan cyclic voltammetry or patch-clamp electrophysiology, which required some visualisations of known markers, but also featured standardized analysis pipelines that had minimal investigator component (see Manuscript Methods). For anatomy experiments, cell selection was performed blindly with respect to astrocyte location.

Reporting for specific materials, systems and methods

We require information from authors about some types of materials, experimental systems and methods used in many studies. Here, indicate whether each material, system or method listed is relevant to your study. If you are not sure if a list item applies to your research, read the appropriate section before selecting a response.

Materials & experimental systems

n/a	Involved in the study	
[ ]	[x]	Antibodies
[x]	[ ]	Eukaryotic cell lines
[x]	[ ]	Palaeontology and archaeology
[ ]	[x]	Animals and other organisms
[x]	[ ]	Clinical data
[x]	[ ]	Dual use research of concern
[x]	[ ]	Plants

Methods

n/a	Involved in the study	
[x]	[ ]	ChIP-seq
[x]	[ ]	Flow cytometry
[x]	[ ]	MRI-based neuroimaging

Antibodies

Antibodies used	Guinea pig anti-S100β (1:1000, 287004, Synaptic Systems; RRID:AB_2620025);<br>Mouse anti-GFAP (1:1000, G6171, Sigma-Aldrich; RRID:AB_1840893);<br>Goat anti-ChAT (1:100; AB114P, Millipore; RRID:AB_2313845);<br>Rabbit anti-NeuN (1:300, ab104255, Abcam; RRID:AB_10716451);<br>Chicken anti-GFP (1:1000, GFP-1010, Lot GFP3717982, Aves; RRID:AB_2307313);<br>Mouse anti-PV (1:1000, P3088, Sigma, RRID:AB_477329)<br>Donkey anti-GFAP antibodies (1:2000; Z0334; Agilent Dako, Santa Clara, United States; RRID:AB_10013382)<br><br>Donkey anti-Guinea pig Alexa Fluor 488 (1:300, 706-545-148, Jackson ImmunoResearch, RRID:AB_2340472);<br>Donkey anti-Goat DyLight 594 (1:150, ab96937, Abcam, RRID:AB_10680873);<br>Donkey anti-Mouse Alexa Fluor 488 (1:300, A-21202, Invitrogen, RRID:AB_141607);<br>Donkey Anti-Guinea Pig Cy3 (1:300, 706-165-148, Jackson ImmunoResearch, RRID:AB_2340460);<br>Donkey anti-Rabbit Alexa Fluor 594 (1:300, 711-585-152, Jackson ImmunoResearch, RRID:AB_2340621);<br>Donkey anti-Chicken Alexa Fluor 488 (1:300, 703-545-155, Jackson ImmunoResearch, RRID:AB_2340375).
Validation	All antibodies have been previously extensively used, validation statements can be found at the relevant suppliers' websites:<br>https://sysy.com/product/287004<br>https://www.sigmaaldrich.com/GB/en/product/sigma/g6171<br>https://www.merckmillipore.com/GB/en/product/Anti-Choline-Acetyltransferase-Antibody,MM_NF-AB144P<br>https://www.abcam.com/products/primary-antibodies/neun-antibody-neuronal-marker-ab104225.html<br>https://www.aveslabs.com/products/anti-green-fluorescent-protein-antibody-gfp<br>https://www.sigmaaldrich.com/GB/en/product/sigma/p3088?srsltid=AfmBOoppOcbMbq4UCTm-KT0dFR9lMo8vc8Ofnj-hAelmEyLF-eooLpq1<br>https://www.antibodyregistry.org/AB_10013382<br><br>https://www.jacksonimmuno.com/catalog/products/706-545-148<br>https://www.abcam.com/index.html?pageconfig=resource&rid=12937<br>https://www.thermofisher.com/antibody/product/Donkey-anti-Mouse-IgG-H-L-Highly-Cross-Adsorbed-Secondary-Antibody-Polyclonal/A-21202<br>https://www.jacksonimmuno.com/catalog/products/706-165-148<br>https://www.jacksonimmuno.com/catalog/products/711-585-152<br>https://www.jacksonimmuno.com/catalog/products/703-545-155

Animals and other research organisms

Policy information about studies involving animals; ARRIVE guidelines recommended for reporting animal research, and Sex and Gender in Research

Laboratory animals	The following animals were used:<br><br>Wildtype C57BL/6J mice<br>Heterozygous ChAT-Cre mice (Jax #006410)<br>Heterozygous Ai32 mice (Ai32 ROSAChR2(H134R)-EYFP, Jax#024109)<br><br>All experiments were performed in group-housed mice, 8-16 weeks of age in both sexes
Wild animals	*Provide details on animals observed in or captured in the field; report species and age where possible. Describe how animals were caught and transported and what happened to captive animals after the study (if killed, explain why and describe method; if released, say where and when) OR state that the study did not involve wild animals.*
Reporting on sex	Animals of both sexes were used throughout the study. For the fast-scan cyclic voltammetry and patch-clamp electrophysiology experiments, we post-hoc examined possible effects of sex, which we did not observe (See Manuscript Supplementary Figures)
Field-collected samples	*For laboratory work with field-collected samples, describe all relevant parameters such as housing, maintenance, temperature, photoperiod and end-of-experiment protocol OR state that the study did not involve samples collected from the field.*
Ethics oversight	All procedures were performed in accordance with the Animals in Scientific Procedures Act 1986 (Amended 2012) with ethical approval from the University of Oxford, and under authority of a Project Licence granted by the UK Home Office.

Note that full information on the approval of the study protocol must also be provided in the manuscript.

Plants

Seed stocks	*Report on the source of all seed stocks or other plant material used. If applicable, state the seed stock centre and catalogue number. If plant specimens were collected from the field, describe the collection location, date and sampling procedures.*
Novel plant genotypes	*Describe the methods by which all novel plant genotypes were produced. This includes those generated by transgenic approaches, gene editing, chemical/radiation-based mutagenesis and hybridization. For transgenic lines, describe the transformation method, the number of independent lines analyzed and the generation upon which experiments were performed. For gene-edited lines, describe the editor used, the endogenous sequence targeted for editing, the targeting guide RNA sequence (if applicable) and how the editor was applied.*
Authentication	*Describe any authentication procedures for each seed stock used or novel genotype generated. Describe any experiments used to assess the effect of a mutation and, where applicable, how potential secondary effects (e.g. second site T-DNA insertions, mosiacism, off-target gene editing) were examined.*
